# A simple cryotransfer method for 3D electron diffraction measurements of highly sensitive samples

**DOI:** 10.1107/S1600576725002456

**Published:** 2025-05-02

**Authors:** Kshitij Gurung, Erik Uran, Klemen Motaln, Petr Brázda, Kristian Radan, Matic Lozinšek, Lukáš Palatinus

**Affiliations:** a Department of Structure Analysis Institute of Physics of the Czech Academy of Sciences Na Slovance 1999/2 Prague 8 18221 Czechia; b Jožef Stefan Institute Jamova cesta 39 1000Ljubljana Slovenia; c Jožef Stefan International Postgraduate School Jamova cesta 39 1000Ljubljana Slovenia; Ecole National Supérieure des Mines, Saint-Etienne, France

**Keywords:** 3D electron diffraction, cryotransfer, xenon fluorides, noble-gas compounds

## Abstract

The accurate characterization of highly sensitive materials using 3D electron diffraction (3D ED) is often challenged by sample degradation caused by exposure to moisture, air, temperature variations and high vacuum during the transfer and introduction into the transmission electron microscope (TEM). A cryogenic sample-transfer protocol is presented here, designed to enable the safe and effective transfer of reactive samples into the TEM, ensuring an inert and moisture-free environment throughout the process. The protocol was validated by redetermining the crystal structures of the moisture-sensitive, strongly oxidizing and highly reactive compounds XeF_2_, XeF_4_ and XeF_2_·XeF_4_ cocrystal. Crystal structures of all three compounds were successfully solved *ab initio* and dynamically refined, yielding results that showed good agreement with the previously reported X-ray and neutron diffraction structures. This approach holds significant promise for advancing the study of other reactive and moisture-sensitive samples, enabling precise structural characterization in cases where traditional TEM sample preparation is unsuitable.

## Abstract 2

A simple protocol for transferring highly moisture-sensitive compounds into a transmission electron microscope for 3D electron diffraction measurements is described, with xenon fluorides as a test case. By maintaining an inert moisture-free environment throughout the transfer step, the integrity of the samples is preserved, thereby rendering the technique applicable to the study of other reactive or strongly oxidizing compounds.

## 1. Introduction

Over the past two decades, 3D electron diffraction (3D ED) has emerged as a powerful and rapidly growing analytical technique for studying the crystal structures of various compounds (Gemmi *et al.*, 2019 ▸). Advances in data collection methods, instrumentation, and computer software solutions and scripts have primarily driven the improvement of the 3D ED technique (Gemmi *et al.*, 2019 ▸; Gruene & Mugnaioli, 2021 ▸; Saha *et al.*, 2022 ▸). Several review articles concerning the handling, data collection and data processing practices for 3D ED measurements of various classes of compounds have been published (Gruene *et al.*, 2021 ▸; Truong *et al.*, 2023 ▸; Yonekura *et al.*, 2023 ▸). Although many samples can be handled straightforwardly, those that are air, moisture, vacuum and/or temperature sensitive are particularly challenging and require specialized sample preparation for measurement using a transmission electron microscope (TEM).

The cryogenic transmission electron microscopy (cryo-TEM) technique, popularly used in structural biology, has been successfully used in studies of air-sensitive battery materials (Wang *et al.*, 2021 ▸; Li *et al.*, 2017 ▸), where Li metal was directly deposited onto the TEM grid electrochemically. However, such a technique may not apply to other reactive samples, such as the noble-gas fluorides, where deposition directly onto the TEM grid is impossible. Recently, a facile sample loading and transfer method to study highly air-sensitive XeF_2_–MnF_4_ adducts using 3D ED was developed (Motaln *et al.*, 2024 ▸).

The present laboratory note provides an in-depth and step-by-step description of the transfer process exemplified on sublimable, moisture-sensitive, highly reactive and strongly oxidizing XeF_2_, XeF_4_ and XeF_2_·XeF_4_ cocrystal. The ED community could benefit from the protocol, which would facilitate the application of 3D ED structural analysis to other extremely sensitive samples.

## 2. Experimental

The synthesis and handling of all compounds was carried out under the exclusion of air and moisture, as xenon fluorides are sensitive to hydrolysis. While XeF_2_ exhibits considerable kinetic stability (Tramšek & Žemva, 2006 ▸), XeF_4_ and XeF_2_·XeF_4_ decompose readily when exposed to moisture and decompose violently upon contact with water. Moreover, XeF_4_ disproportionates upon hydrolysis to form extremely explosive and highly shock-sensitive XeO_3_ (Chernick, 1966 ▸; Holloway, 1967 ▸; Goettel & Schrobilgen, 2016 ▸). Additionally, all compounds used in this study liberate HF as a hydrolysis product, necessitating the use of proper protective equipment. The appropriate first aid kit must be readily accessible during all procedures (Segal, 2000 ▸). The use of small quantities throughout the whole procedure is strongly advised.

### 2.1. Synthesis of XeF_2_, XeF_4_ and XeF_2_·XeF_4_ cocrystal

XeF_2_ was synthesized via a UV-aided photochemical reaction (Šmalc *et al.*, 1992 ▸) from gaseous xenon (Messer, 99.99%) and fluorine (Solvay Fluor, 98–99%). XeF_4_ was prepared by room-temperature fluorination of XeF_2_ in anhydrous HF with elemental fluorine in the presence of a UV light source (Tavčar & Žemva, 2009 ▸). The purity of the compounds was confirmed by Raman spectroscopy and powder X-ray diffraction (PXRD).

The cocrystal XeF_2_·XeF_4_ was synthesized in the solid state from XeF_2_ and XeF_4_ using a recently developed mechano­chemical approach, which will be fully described in an ensuing paper. A small amount of the resulting fine white powder was loaded into a fluorine-passivated quartz capillary, and it was shown to be pure by Raman spectroscopy and PXRD.

### 2.2. Cryotransfer apparatus

The following apparatus is required for the cryotransfer procedure:

(i) A cryotransfer holder with a TEM grid inserted.

(ii) An air-tight sleeve for the holder.

(iii) An acrylic glovebox with a moisture-level sensor.

(iv) An insulating polystyrene box of suitable dimensions to fit in the antechamber of the glovebox.

(v) About 2 l of liquid N_2_.

**Figure 1:**
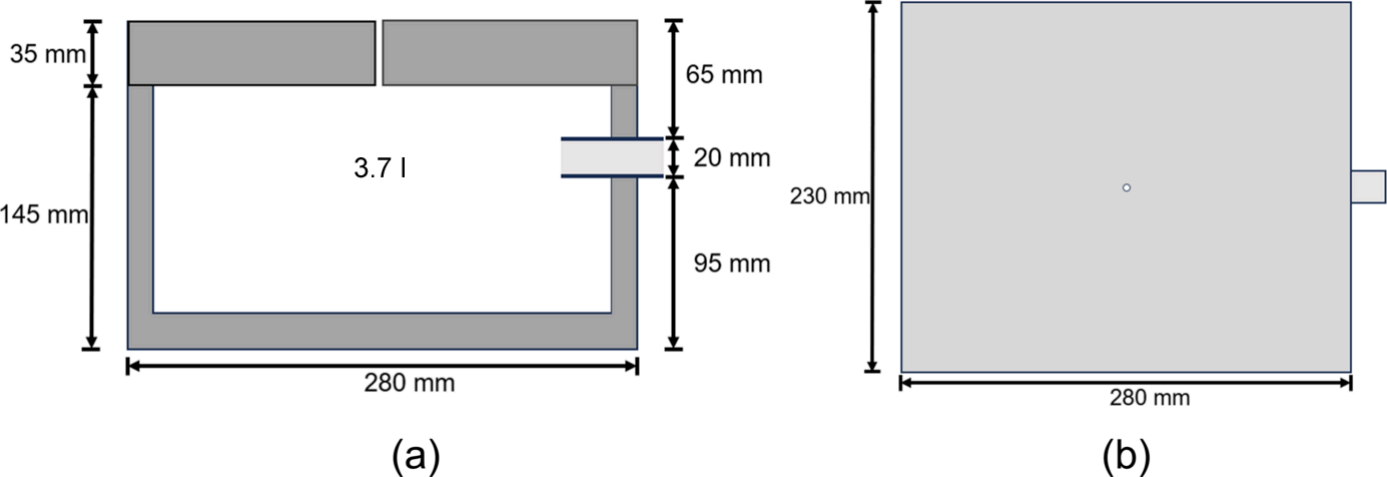
Schematic diagrams of the modified polystyrene box for the cryotransfer in the glovebox. (*a*) Sectional view from the side and (*b*) top view. Images are drawn to scale.

A polystyrene box with outer dimensions of 280 × 230 × 180 mm and a 3.7 l internal volume was used for the cryotransfer of the sensitive samples (Fig. 1 ▸). A 20 mm diameter hole was cut into one side of the box to accommodate a PVC tube (outer diameter 20 mm, inner diameter 14 mm). The tube allows the tip of the cryotransfer holder (referred to simply as the ‘holder’ henceforth) to be inserted into the box, ensuring that the O-ring on the holder seals securely against the tube. The box has a lid with an opening cut out, to allow venting when the box is filled with liquid N_2_.

### 2.3. The glovebox setup

**Figure 2:**
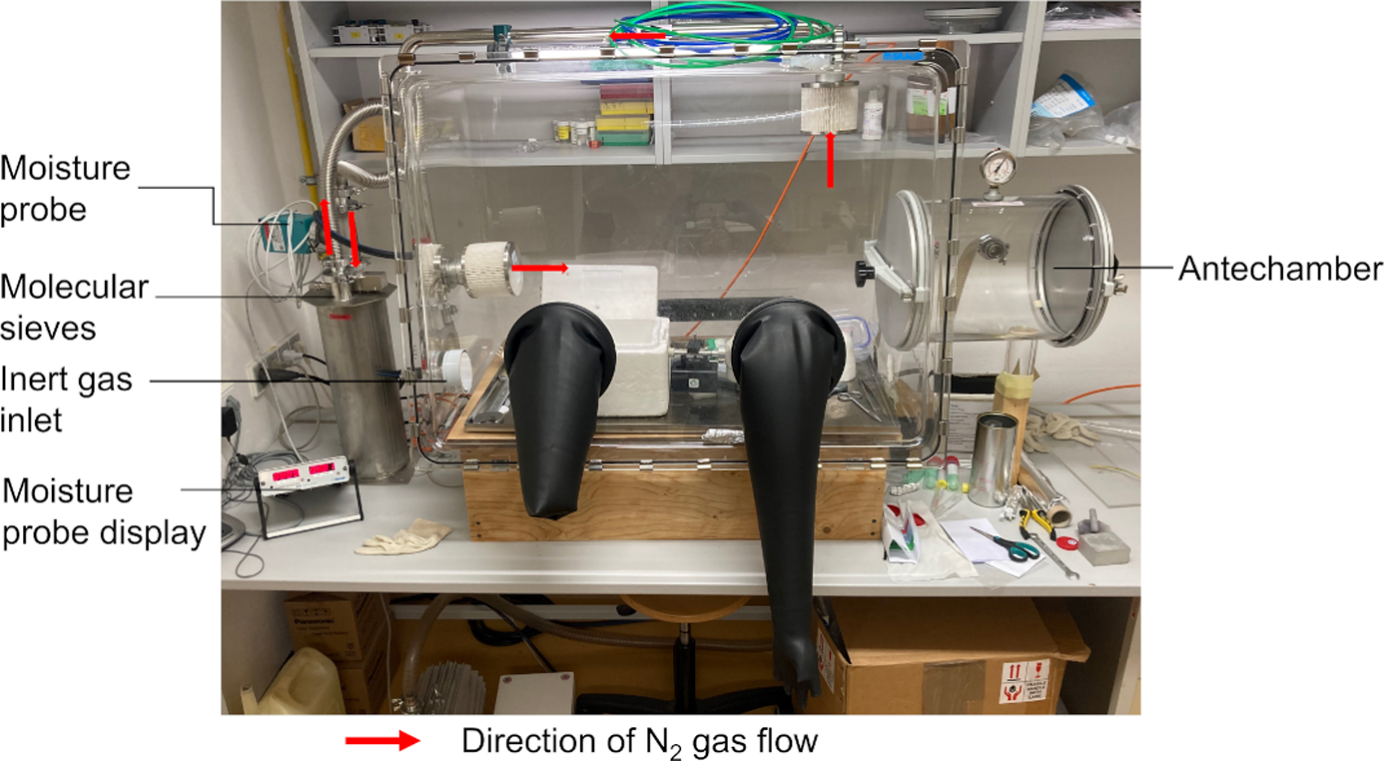
Glovebox used for loading the sample onto the holder, with the polystyrene box and TEM holder inside.

The glovebox setup (Fig. 2 ▸) consists of an acrylic glovebox by MBRAUN filled with N_2_ gas. The humidity in the box is kept to a minimum (≤1 ppm) by circulating the atmosphere in the glovebox through a column with molecular sieves. The H_2_O level in the box is monitored by a moisture probe (MB-NO-SE1 by MBRAUN). Although the probe indicated that no moisture entered the glovebox, it is still advisable to take precautions by using a dedicated glovebox for this procedure, while storing larger amounts of air-sensitive samples and reagents in a separate glovebox.

### 2.4. The cryotransfer process

**Figure 3:**
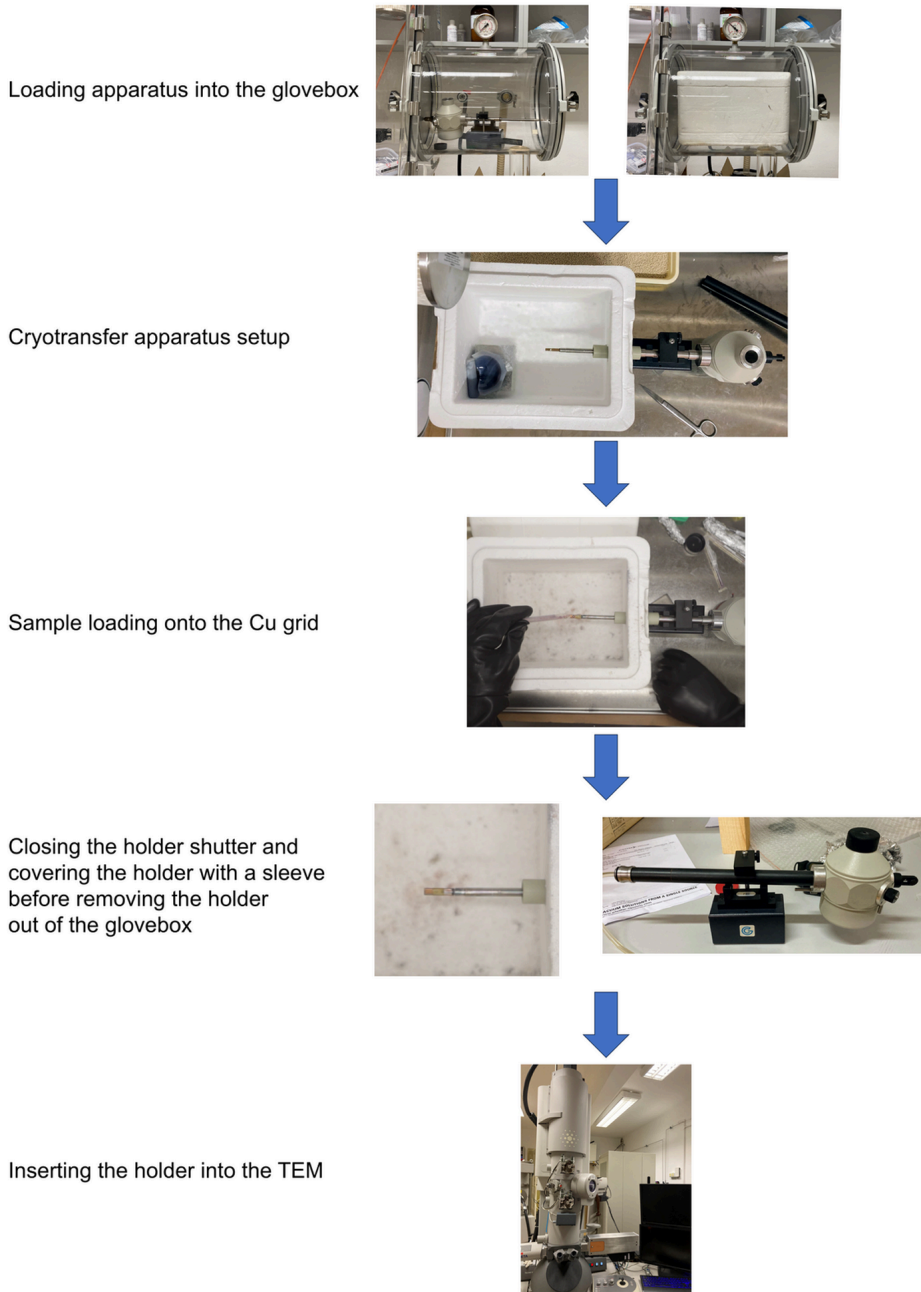
Flow diagram of the cryotransfer process using the cryotransfer holder, polystyrene box, liquid nitro­gen and glovebox.

The cryotransfer process consists of three steps: (i) loading the apparatus into the glovebox, (ii) loading the sample onto the holder and (iii) the transfer of the holder into the TEM (Fig. 3 ▸, and video in the supporting information).

#### 2.4.1. Loading the cryotransfer apparatus into the glovebox

A holder (Gatan model 914, high-tilt liquid nitro­gen cryotransfer tomography holder), loaded with a holey carbon-coated copper TEM grid, was transferred into the glovebox after three pump/refill cycles in the antechamber (pumped down to ∼0.1 atm). The polystyrene box filled with liquid N_2_ and covered with its lid was then transferred into the glovebox. The antechamber was only pumped to 0.5 atm pressure and refilled three times before transferring the box into the glovebox. The lack of white-colored mist coming out of the vent of the polystyrene box during the final evacuation cycle is a good indication of the low humidity in the antechamber. Additionally, the low moisture level in the polystyrene box can be verified by the clear appearance of the surface of the liquid N_2_ once the box is transferred into the glovebox (see the video in the supporting information).

#### 2.4.2. Loading the sample onto the holder

The tip of the holder was inserted into the polystyrene box through the side opening. Using a polystyrene cup, some of the liquid N_2_ was dispensed from the box into the dewar of the holder. The holder was allowed to cool for 10–15 min, ensuring it reached a temperature below −160 °C. At this temperature, the holder is very susceptible to the deposition of ice, even if only trace amounts of humidity are present in the glovebox. The advantage of this setup is that the tip of the holder in the box is surrounded by N_2_ gas vaporizing from the liquid N_2_, effectively shielding the tip from any residual humidity in the glovebox. Once the holder was cooled, a small amount of sample was dispensed onto the grid and the holder was then gently tapped to remove excess sample. By this point, the temperature was sufficiently low to prevent the sample from reacting with the grid or holder.

#### 2.4.3. Loading the holder into the TEM

**Figure 4:**
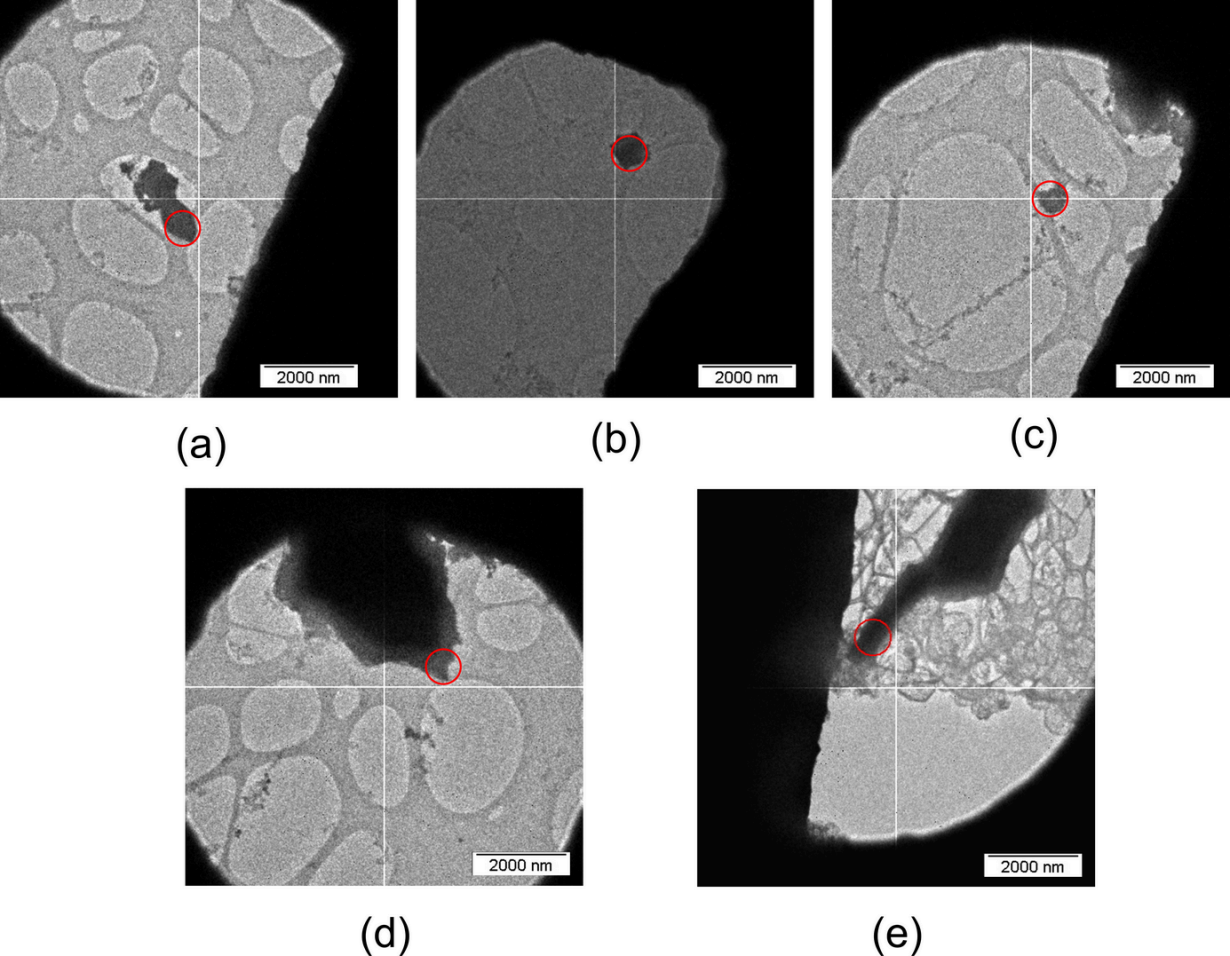
TEM images of crystals of (*a*) XeF_2_, (*b*), (*c*) XeF_4_, and (*d*), (*e*) XeF_2_·XeF_4_ used for 3D ED measurements. The red circles denote the beam sizes used for 3D ED data collection.

First, the shutter of the holder was closed, and then the holder was removed from the polystyrene box. Immediately, the tip was covered by a sleeve. The sleeve isolates the tip of the holder and allows safe removal of the holder from the glovebox. Since the holder must be rotated for insertion into the microscope, the liquid N_2_ was first dispensed from the dewar, and the holder was swiftly removed from the sleeve and inserted into the TEM. Immediately afterwards, the dewar of the holder was refilled with liquid N_2_. During the whole process, the tip of the holder with the closed shutter was exposed to the atmosphere for a maximum of 2 to 4 s and the temperature remained below −140 °C. Because of the low temperature, the sample did not react with air or the TEM grid during this step. The holder’s shutter also protects the sample from direct contact with air throughout the insertion procedure, as demonstrated by the fact that minimal ice formation is observed on the grids (Fig. 4 ▸). Maintaining cold temperatures is also necessary because XeF_2_, XeF_4_ and XeF_2_·XeF_4_ exhibit vapor pressure at room temperature (Schreiner *et al.*, 1968 ▸) and can easily be sublimed away under a dynamic vacuum.

### 2.5. 3D ED measurements

The general microscope information and experimental conditions for the 3D ED measurements are provided in Table 1 ▸. The crystallites of the three different chemical species that were measured in this work, XeF_2_ [Fig. 4 ▸(*a*)], XeF_4_ [Figs. 4 ▸(*b*) and 4 ▸(*c*)] and XeF_2_·XeF_4_ [Figs. 4 ▸(*d*) and 4 ▸(*e*)], were typically smaller than 1 µm. One of the main advantages of 3D ED is that it allows single-crystal diffraction measurements and crystal structure determination to be performed on submicrometre-sized crystals. For both XeF_4_ and XeF_2_·XeF_4_, two data sets from separate crystallites were merged to obtain more complete data sets for the structure solution and refinement.

### 2.6. 3D ED data processing

The *PETS2* program (Palatinus *et al.*, 2019 ▸) was used for indexing, determination of lattice parameters and peak integration of the diffraction patterns. The processed data were imported into *JANA2020* (Petříček *et al.*, 2023 ▸) and the crystal structures were determined *ab initio* using *SIR2014* (Burla *et al.*, 2015 ▸). The resulting structures were initially refined kinematically and then refined dynamically in *JANA2020* (Palatinus *et al.*, 2015 ▸; Klar *et al.*, 2023 ▸). For XeF_2_ and XeF_4_, all the atoms were refined anisotropically. For the XeF_2_·XeF_4_ cocrystal, except for two fluorine atoms bonded to Xe^IV^, which were refined isotropically, all the other atoms were refined anisotropically. Refinement information and results are listed in Table 2 ▸.

## 3. Experimental results and discussion

**Figure 5:**
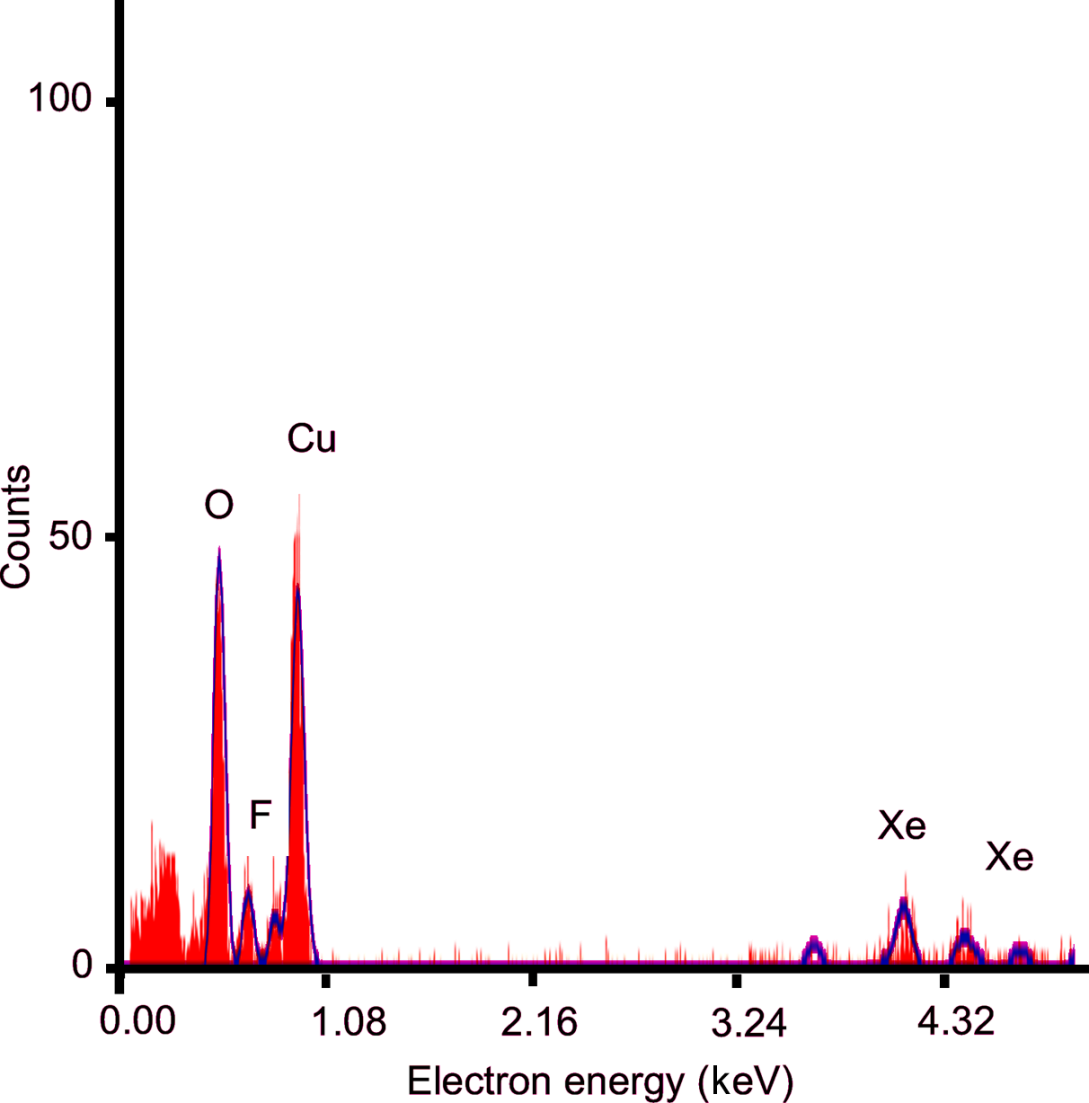
EDS spectrum of an XeF_2_·XeF_4_ crystal with fitted peaks for Xe, F, O and Cu, highlighted in blue, indicating their presence in the analyzed region. The presence of Xe demonstrates that the sample transfer was successful.

**Figure 6:**
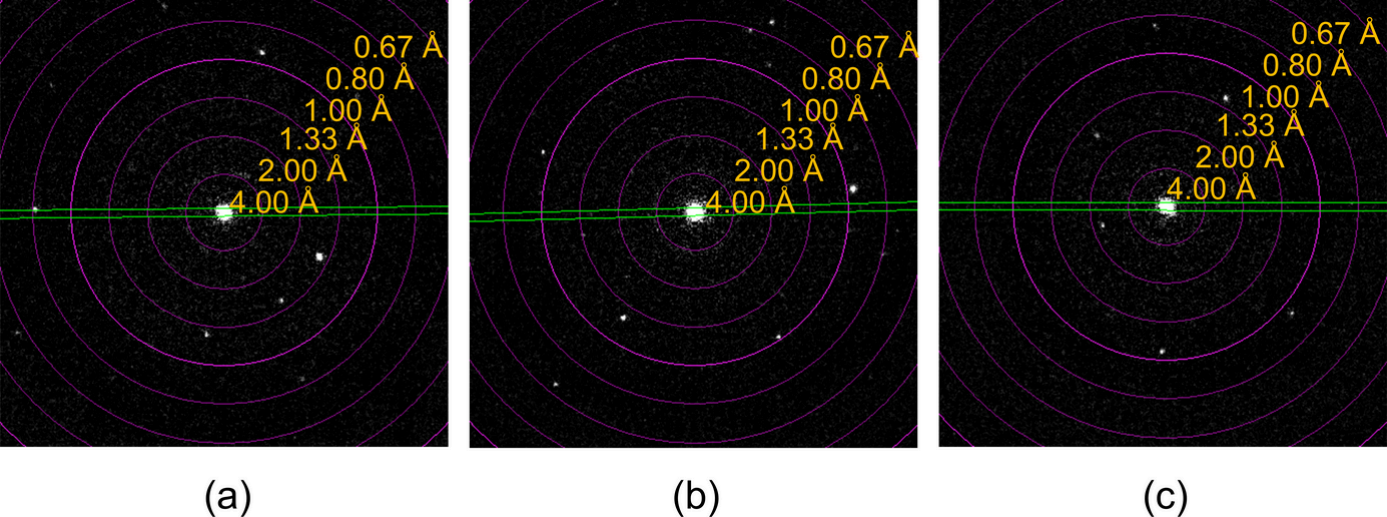
Starting frames of the 3D ED data collection on (*a*) XeF_2_, (*b*) XeF_4_ and (*c*) XeF_2_·XeF_4_ that show high-resolution reflections (*d*_min_ < 0.83 Å).

The presence of both xenon and fluorine in the energy-dispersive X-ray spectroscopy (EDS) spectrum (Fig. 5 ▸) is a good indication that the compounds did not hydrolyze during the employed transfer procedure, and the high-resolution diffraction patterns (Fig. 6 ▸) with resolutions *d*_min_ < 0.80 Å for all three phases indicate that the compounds have retained their crystallinity. All measured crystals were beam stable at a flux density of 0.0460 e^−^ Å^−2^ s^−1^ during the whole experiment and maintained their resolutions better than 1.0 Å throughout the data collection.

**Figure 7:**
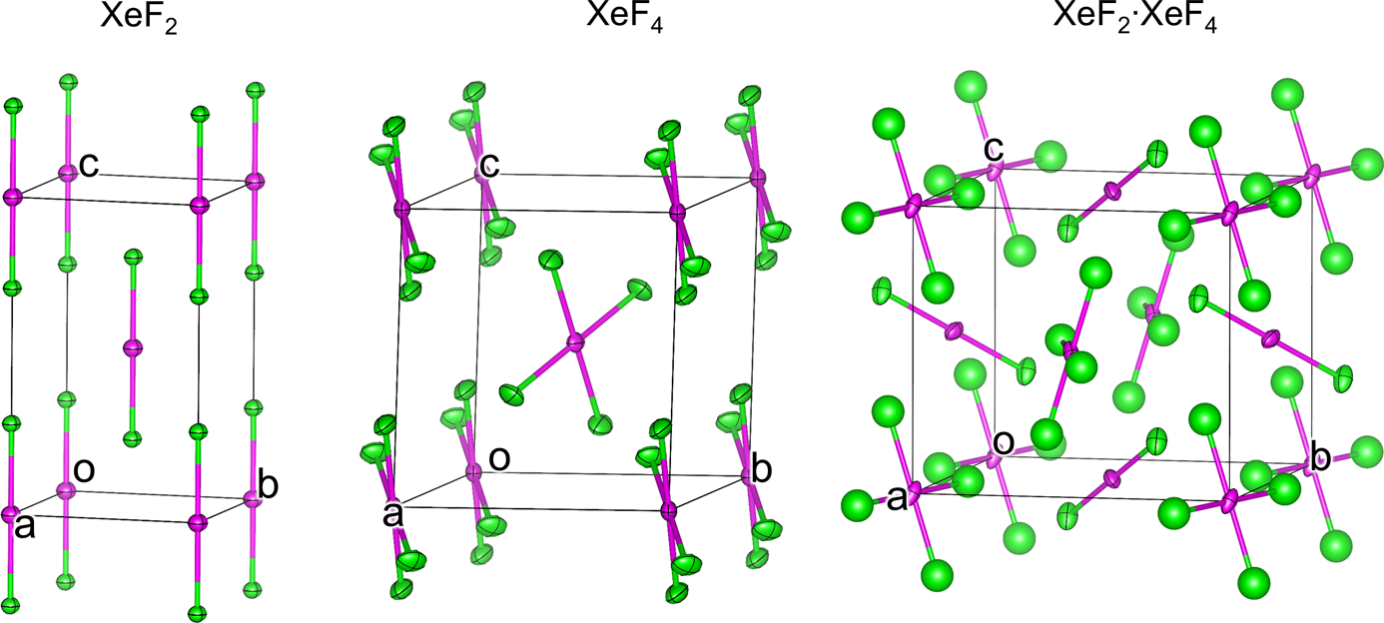
Crystal structures of XeF_2_, XeF_4_ and XeF_2_·XeF_4_, solved and dynamically refined from 3D ED data. Displacement ellipsoids are drawn at the 50% probability level.

Crystal structures of all three compounds were successfully solved *ab initio*. The dynamically refined structures are depicted in Fig. 7 ▸. For the XeF_2_·XeF_4_ cocrystal, the data quality only allowed isotropic refinement of the fluorine atoms bonded to Xe^IV^. These fluorine atoms exhibit comparatively large anisotropic displacement parameters (ADPs), leading to the Xe—F bond lengths differing from the reported values (Bortolus *et al.*, 2021 ▸) by up to 0.052 Å and the F—Xe^IV^—F angle differing from 90° by 2.1° (Table 2 ▸). While these values are larger than the typical deviations in dynamic refinement, they are within the expected range, especially given the limited quality of the data (Klar *et al.*, 2023 ▸). Overall, the results from the 3D ED experiments for all three compounds (Table 2 ▸) are in good agreement with the literature single-crystal X-ray diffraction (Ibers & Hamilton, 1963 ▸; Elliott *et al.*, 2010 ▸; Burns *et al.*, 1965 ▸; Siegel & Gebert, 1963 ▸; Templeton *et al.*, 1963 ▸; Bortolus *et al.*, 2021 ▸; Burns, 1963 ▸) or single-crystal neutron diffraction (Levy & Agron, 1963 ▸) results, thus showing that this cryotransfer method was successful for these moisture-sensitive, highly reactive and strongly oxidizing compounds.

Mechanochemistry is an emerging green, sustainable and low-cost synthesis technique (Belak Vivod *et al.*, 2024 ▸; Do & Friščić, 2017 ▸), but the structural analysis of the resulting powdered product is typically hampered by submicrometre-sized crystallites, thus rendering single-crystal X-ray diffraction unsuitable. The present successful 3D ED structural determination of mechanochemically synthesized XeF_2_·XeF_4_ demonstrates the robustness of the described sample-handling protocol and confirms the capability of 3D ED for direct structural elucidation of mechanochemical products (Sala *et al.*, 2024 ▸).

## 4. Conclusion

In conclusion, a novel and cost-effective cryotransfer protocol for handling reactive moisture-sensitive samples for 3D ED studies has been developed, and tested on highly reactive xenon fluorides. Submicrometre-sized crystallites of XeF_2_, XeF_4_ and XeF_2_·XeF_4_ were successfully transferred onto a cryo-holder and introduced into the TEM, single-crystal 3D ED data were readily collected, and the crystal structures were determined. The obtained structural parameters are consistent with literature values, indicating that the integrity of the samples was preserved. This work paves the way for applying 3D ED methodologies to a wide range of sensitive and reactive samples.

## Supplementary Material

Crystal structure: contains datablock(s) global, I, global_1, I_1, global_2, I_2. DOI: 10.1107/S1600576725002456/nb5398sup1.cif

Structure factors: contains datablock(s) I. DOI: 10.1107/S1600576725002456/nb5398Isup2.hkl

Structure factors: contains datablock(s) II. DOI: 10.1107/S1600576725002456/nb5398IIsup3.hkl

Structure factors: contains datablock(s) III. DOI: 10.1107/S1600576725002456/nb5398IIIsup4.hkl

Video showing use of the apparatus. DOI: 10.1107/S1600576725002456/nb5398sup5.mp4

CCDC references: 2431936, 2431937, 2431938

## Figures and Tables

**Table:** 

Microscope	FEI Tecnai G^2^ 20 transmission electron microscope
Radiation source	LaB_6_
Detector (type)	Medipix 3 hybrid pixel detector ASI CheeTah (512 × 512 pixels, 24-bit dynamic range)
Accelerating voltage (kV)	200
Wavelength, λ (Å)	0.02508
Probe type	Microdiffraction
Detector pixel size (µm)	55 × 55
Measurement temperature, *T* (K)	100
Flux density (e^−^ Å^−2^ s^−1^)	0.0460
3D ED collection method	Continuous-rotation data collection

**Table:** 3D ED experimentation information.

	XeF_2_	XeF_4_ (two crystals)	XeF_2_·XeF_4_ (two crystals)
Exposure time (ms per frame)	386	386, 386	386, 405
Beam diameter (nm)	800	800, 800	800, 800
Tilt angles, α_min_, α_max_, Δα (°)	−9.72, 59.48, 0.30	0.63, 39.02, 0.30	−29.82, 30.37, 0.40
		0.18, 44.72, 0.30	−50.51, 52.38, 0.30

**Table 2:** Crystal structure and post-refinement information

	XeF_2_	XeF_2_†	XeF_4_	XeF_4_‡	XeF_2_·XeF_4_	XeF_2_·XeF_4_‡
Formula unit, *Z*	2	2	2	2	2	2
Space group	*I*4/*mmm*	*I*4/*mmm*	*P*2_1_/*n*	*P*2_1_/*n*	*P*2_1_/*c*	*P*2_1_/*c*
*T* (K)	100	100	100	100	100	100
*a* (Å)	4.2249 (10)	4.2188 (7)	5.0148 (8)	4.9474 (3)	6.5273 (8)	6.4389 (5)
*b* (Å)	4.2249 (10)	4.2188 (7)	5.7392 (15)	5.7792 (4)	7.2727 (6)	7.2692 (6)
*c* (Å)	6.9526 (18)	6.991 (2)	5.9043 (14)	5.7872 (4)	6.3644 (10)	6.2293 (5)
β (°)	90	90	100.866 (15)	100.279 (2)	92.951 (11)	92.577 (3)
*V* (Å^3^)	124.10 (5)	124.43 (5)	166.88 (7)	162.812 (19)	301.72 (7)	291.27 (4)
Apparent mosaicity (°)	0.190		0.108, 0.003		0.083, 0.164	
Completeness (%)	100.0		78.7		87.1	

Bond lengths (Å)						
Xe^II^—F	1.992 (8)	1.999 (4)			1.975 (4)	1.9940 (9)
Xe^IV^—F			1.964 (6)	1.9509 (6)	1.955 (5)	1.937 (1)
			1.949 (6)	1.9449 (6)	1.889 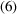	1.9412 (9)

Bond angles (°)						
F—Xe^II^—F	180.0	180.0			180.0	180.0
F—Xe^IV^—F			90.7 (2)	90.26 (3)	92.6 (3), 180.0	90.47 (5), 180.0

Dynamic refinement results						
Maximum resolution, *d*_min_ (Å)	0.71		0.71		0.71	
*N*_obs_, *N*_all_	434, 434		1145, 2116		1382, 4515	
Parameters	44		111		132	
*R*_obs_, *wR*_obs_ (%)	12.30, 15.85		10.73, 11.96		8.02, 7.23	
*R*_all_, *wR*_all_ (%)	12.30, 15.85		13.41, 12.21		19.43, 8.46	

Computer programs used: *PETS2* (Palatinus *et al.*, 2019 ▸), *JANA2020* (Petříček *et al.*, 2023 ▸), *SIR2014* (Burla *et al.*, 2015 ▸), *Mercury 4.0* (Macrae *et al.*, 2020 ▸).

† Data from Elliott *et al.* (2010 ▸).

‡ Data from Bortolus *et al.* (2021 ▸).
